# Management of Common Toxicities in Metastatic NSCLC Related to Anti-Lung Cancer Therapies with EGFR–TKIs

**DOI:** 10.3389/fonc.2014.00238

**Published:** 2014-09-16

**Authors:** Barbara Melosky, Vera Hirsh

**Affiliations:** ^1^Medical Oncology, British Columbia Cancer Agency – Vancouver Centre, Vancouver, BC, Canada; ^2^Department of Medical Oncology, McGill University Health Centre, Royal Victoria Hospital, Montreal, QC, Canada

**Keywords:** tyrosine kinase inhibitor, EGFR, adverse event management, diarrhea, rash, stomatitis/mucositis, paronychia

## Abstract

Tyrosine kinase inhibitors (TKIs) against the epidermal growth factor receptor (EGFR) are the standard of care treatment in non-small cell lung cancer (NSCLC). TKIs are used first line in EGFR mutation-positive NSCLC; erlotinib is the only TKI approved for subsequent lines of treatment in EGFR wild-type NSCLC. As promising as TKIs are in helping patients avoid some of the side effects of traditional cytotoxic chemotherapy, they do come with a variety of side effects. This article will describe the most common adverse events associated with the epidermal EGFR family of TKIs including diarrhea, rash, mucositis, and paronychia. The objective of this paper is to provide simple guidelines to assist oncologists in managing these common toxicities. As patient survival is often directly correlated with successful therapeutic drug delivery, the management of TKI-induced adverse events ensures proper treatment and may avoid discontinuation or reduction of the therapeutic.

## Introduction

Tyrosine kinase inhibitors (TKI) are a relatively new class of targeted therapeutics used to treat a range of diseases and disorders, primarily cancers. The first TKIs, described in 1988, specifically inhibited the epidermal growth factor receptor (EGFR) cascade (1). This unique class of orally administered small molecule therapeutics has found their way into the standard of care treatment in almost all types of malignancy including non-small cell lung cancer (NSCLC). Many new EGFR–TKIs will continue to emerge, and the number of TKls in use will continue to expand.

Tyrosine kinase inhibitors may help patients avoid some of the side effects of traditional cytotoxic chemotherapy, where toxicities usually involve bone marrow involvement. However, as promising as the TKIs are, they do come with a variety of side effects. These gastroenterologic side effects, such as diarrhea and mucositis, and cutaneous side effects, such as rash and paronychia, deserve attention as successful management can extend the time that the patient is on therapy. To date, there have been few clinical studies conducted to study these side effects. The objective of this paper is to provide simple guidelines to assist oncologists in managing these common toxicities. While the use of EGFR–TKIs can also have more severe adverse events (SAE) including ocular disorders, interstitial lung disease, or hepatotoxicity, these SAE are uncommon and fall beyond the scope of this paper.

## Diarrhea

Diarrhea is one of the most frequent adverse events of EGFR–TKI therapy. The gastrointestinal tract expresses EGFR on cells of epithelial origin.

### Causes and incidence

Epidermal growth factor receptor–tyrosine kinase inhibitors induced diarrhea is thought to result from excess chloride secretion that causes a secretory form of diarrhea (2). Severe diarrhea can result in fluid and electrolyte loss, which then can lead to dehydration, electrolyte imbalances, and renal insufficiency. Alterations in gastrointestinal transit and digestion can lead to nutritional deficiencies that can negatively impact the quality of life (QOL) of patients (3).

The incidence of diarrhea with EGFR–TKI treatment in phase III clinical trials ranges from 27 to 87% with up to 25% of patients experiencing SAEs (Table 1).

**Table 1:** **Incidence of diarrhea with EGFR–TKIs in NSCLC clinical trials**.

EGFR–TKI	Description	Grade (%)
		All ≥3
Erlotinib 150 mg	All studies	(10–69) (0–17)
	Phase III studies	(40–68) (2–12)
Gefitinib 250 and 500 mg	All studies	(27–75) (0–25)
	• 250 mg	(27–58) (0–10)
	• 500 mg	(51–75) (5–25)
	Phase III studies	(27–69) (3–25)
	• 250 mg	(27–58) (3–10)
	• 500 mg	(51–69) (12–25)
Afatinib 40 and 50 mg	All studies	(67–100) (0–33)
	• 40 mg	(67–97) (0–7)
	• 50 mg	(87–100) (17–33)
	Phase III studies	
	• 50 mg	(87–17)
Dacomitinib 15, 30, and 45 mg	All studies (phase II)	(77–97) (0–15)
	• 30 mg	(77–0)
	• 45 mg	(81–97) (13–15)

*Adapted from Hirsh (10)*.

### Grading and Assessment of Diarrhea

The severity of diarrhea is graded using the National Cancer Institute’s CTCAE (Table 2). These criteria (grades) do not provide a complete assessment, and additional information should be obtained from the patient evaluation. It is important to rule out other possible causes of diarrhea. These include medications such as laxatives, stool softeners, antibiotics, or antacids; dietary factors such as excess consumption of fiber or dairy products, greasy foods; comorbid infections such as intestinal obstruction, fecal impaction, and surgeries (short bowel or gastrectomy); or radiation toxicity.

**Table 2:** **US National Cancer Institute grading for diarrhea**.

Grade 1	Grade 2	Grade 3	Grade 4	Grade 5
An increase of <4 stools over baseline, per day	An increase of 4–6 stools over baseline, per day	An increase of 7 or more stools over baseline per day	Life-threatening consequences	Death
		Incontinence	Urgent intervention indicated	
		Hospitalization indicated	
		Limits self-care activities of daily living	

*Adapted from the Common Terminology Criteria for Adverse Events (15)*.

Laboratory investigations include a complete blood count and differential to rule out neutropenia, blood tests to assess renal function, and electrolyte abnormalities and a stool culture or *Clostridium difficile* toxin screen to check for bacterial pathogens. To rule out co-existing disorders such as bowel obstruction or perforation, abdominal radiography, endograph endoscopy, or biopsy might need to be performed. Duration of diarrhea, stool characteristics, and co-existing symptoms should also be obtained from the patient (3, 4).

### Management recommendations for diarrhea

Patients should be advised to immediately report any symptoms of diarrhea, so they can be managed early and effectively. Patients have to understand the importance of avoiding/preventing dose reductions or discontinuation of EGFR–TKIs.

Dietary changes and over-the-counter anti-diarrheal medications can generally be used to manage EGFR–TKI induced diarrhea. This management is identical to that of chemotherapy-induced diarrhea (3–5).

Patients who experience diarrhea should avoid greasy, spicy, and fried foods as they can exacerbate the symptoms. Until symptoms start to resolve, patients can eat a diet of bananas, rice, apple sauce, and toast (BRAT) and avoid foods that may increase abdominal cramping and bloating such as Brussels sprouts, cabbage, and broccoli. When symptoms start to improve, foods such as eggs, pasta, and skinless chicken can be added. Patients should drink 3–4 l of fluid to prevent dehydration. Prolonged diarrhea may cause diminished lactase activity resulting in lactose intolerance thus milk products should be avoided for about a week following diarrhea (3–5).

**Figure 1:**
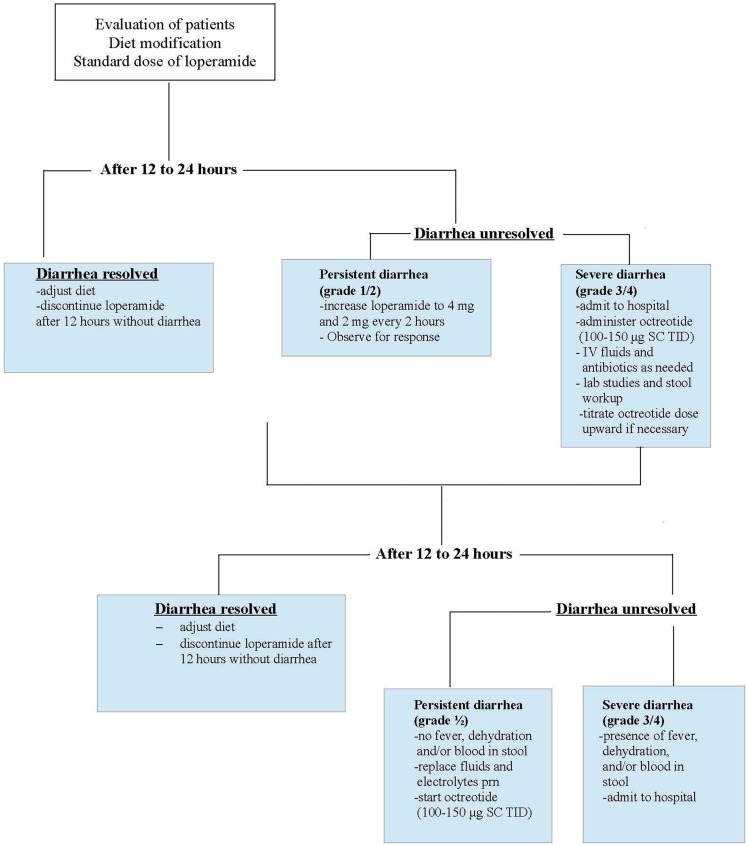
**Management of diarrhea induced by chemotherapy or EGFR–TKIs**. IV, intravenous, SC, subcutaneous, TID, three times daily. Adapted from Hirsh (10).

The pharmacologic management of diarrhea is generally limited to over-the-counter loperamide (Figure 1). After the first diarrhea, patients should start 4 mg of loperamide followed by 2 mg after each loose stool or every 4 h to a maximum daily dose of 20 mg. If symptoms persist for more than 24 h, the dose of loperamide can be increased to 4 mg followed by 2 mg every 2 h. If 12 h have passed without diarrhea, loperamide can be stopped (3–6).

Epidermal growth factor receptor–tyrosine kinase inhibitors cessation is required for grade 3 or 4 diarrhea and then restart EGFR–TKI at a lower dose once the severe symptoms have subsided. Use of octreotide is generally for chemotherapy-induced diarrhea and there is no evidence to support its use in the EGFR–TKI setting.

## Rash

Cutaneous side effects of EGFT–TKIs can include skin rashes of many different types and severity. The rash is very acne-like in appearance, and is accurately described as a papulopustular eruption. Other descriptions include the terms acneiform skin reaction, acneiform rash, acneiform follicular rash, acne-like rash, maculopapular skin rash, and amonomorphic pustular lesions. The EGFR–TKI induced rash most often appears on the face and chest, but can be more widespread. The rash may be triggered by sun exposure (7). Dry skin, pruritus, ocular, and hair changes are also common.

### Causes and incidence

Epidermal growth factor receptor is expressed in the basal layer of the epidermis, and its normal physiological roles include stimulation of epidermal growth, inhibition of differentiation, and acceleration of wound healing. As the name of this receptor is “epidermal,” it is no surprise that inhibitor toxicity may include the epidermis. Pathophysiological effects of EGFR inhibition include impaired growth and migration of keratinocytes, and the expression of inflammatory chemokines by these cells, which results in inflammatory cell recruitment (8). Not surprisingly, a histologic analysis demonstrates a mixed inflammatory infiltrate in the upper areas of the skin. This inflammation and subsequent cutaneous injury accounts for many of the symptoms observed in patients being treated with this class of TKI, including tenderness, papulopustules, and periungual inflammation (9).

There are several phases to the cutaneous manifestations. In the first week of TKI treatment, patients often experience sensory disturbances, erythema, and edema. In the second week of TKI treatment, patients experience papulopustular eruptions, followed by crusting in week 4. In the 4–6 weeks following, a background of erythema and dry skin can be seen in areas previously affected by the papulopustular eruption (8).

Incidence of all grades of rash in phase 3 clinical trials varies from 37 to 78% (10). Grade 3 rash ranges from 3.1 to 16.2% depending on the trial (Table 3) (11–14).

**Table 3:** **Rate of grade 3 rash, *Paronychia* and *Stomatitis/mucositis* observed in trials**.

Grade 3/4 adverse events	EGFR–TKI and trial			
	Gefitinib	Erlotinib	Afatinib	Afatinib
	IPASS (3) (*N* = 607) (%)	EURTAC (4) (*N* = 84) (%)	LUX-Lung 3 (5) (*N* = 229) (%)	LUX-Lung 6 (6) (*N* = 239) (%)
Rash/acne	3.1	13	16.2	14.6
Stomatitis/mucositis	0.2	NR	8.7	5.4
Paronychia	0.3	NR	11.4	NR

*NR: not reported*.

### Management recommendations for skin rash

Patient education is a very important aspect of management, and several important points need to be communicated and emphasized. First of all, the rash is not contagious; the skin toxicity is not infectious, but inflammatory. Secondly, this skin rash is not acne, and so patients should be strongly discouraged from treating the rash with over-the-counter acne medications, as acne medication is very drying and will exacerbate the pruritus. Third, as dry skin is almost universally experienced by patients taking this medication, they should be instructed to use an alcohol-free emollient cream applied twice daily, preferably to their entire body. Finally, since sun exposure may aggravate the pruritus, patients are advised to avoid sun exposure, and a broad spectrum sunscreen is strongly recommended (9).

The specific treatment algorithms for rashes caused by EGFR inhibitors vary widely throughout the different centers that use these agents in their clinics. Nonetheless, some basic principles may apply to all situations. Early treatment of rash can prevent symptoms from becoming worse, so clinicians are advised to assess patients weekly, and intervene when the first symptoms of rash appear. Management strategies for EGFR–TKI induced rash are shown in Table 4.

**Table 4:** **BCCA management guidelines for EGFR–TKI induced rash**.

Grade	Toxicity	EGFR inhibitor
1	Macular or papular eruption or erythema with no associated symptoms	Maintain dose level of TKI Consider clindamycin 2% and hydrocortisone 1% in a lotion to be applied topically BID as needed
2	Macular or papulopustular eruption or erythema with pruritus or other symptoms that are tolerable or interfere with daily life	Maintain dose level of TKI Consider clindamycin 2% and hydrocortisone 1% in a lotion to be applied topically BID as needed +minocycline 100 mg PO BID for 1–2 weeks or longer as needed
3	Severe, generalized erythroderma, or macular, popular or vesicular eruption	Withhold EGFR TKI for 10–14 days When improvement to grade 2 or less, continue at 50% of original dose If toxicities do not worsen, escalate by 25% increments of original dose until starting dose is reached If no improvement, discontinue Continue treatment with clindamycin 2% and hydrocortisone 1% in a lotion to be applied topically BID as needed +minocycline 100 mg PO BID for 1 to 2 weeks or longer as needed
4	Generalized exfoliative, ulcerative, or blistering skin toxicity	Discontinue treatment

*Adapted from the management guidelines utilized in the BC Cancer Agency (BCCA) Oncology Department*.

Mild reactions (NCI-CTC grade 1) (15) are generally localized with no associated physical symptoms. Treatment options include topical low–medium potency corticosteroids. Other options include the addition of clindamycin 1% gel to hydrocortisone 1% and the use of oral semisynthetic tetracyclines (i.e., doxycycline or minocycline). The EGFR inhibitor should be continued while the rash is being treated.

Moderate reactions (NCI-CTC grade 2) (15) are more severe and can include symptoms such as tenderness or itching. The recommended treatment is hydrocortisone 1 or 2.5% cream ± clindamycin 1% cream, as well as a 4-week course of an oral tetracycline antibiotic, such as doxycycline 100 mg daily or minocycline 100 mg twice daily. Minocyline can cause nausea in a small percentage of patients, and reduction to 100 mg daily may be better tolerated. As the rash from the EGFR–TKI may wax and wane, the treatment may need to be repeated at several intervals.

Severe reactions (NCl-CTC grade 3) (15) are generalized with major symptoms affecting activities of daily living and are intolerable to the patient. Though histological findings suggest that the papulopustular eruption has an inflammatory component, the use of topical oral corticosteroids is based on empirical data. A temporary 7–10 days discontinuation of the TKI involved is recommended, with subsequent reintroduction at a lower dose according to the product monograph. Treatment with both a steroid cream and oral tetracycline as per moderate rash is encouraged during the interruption period. When treatment is reintroduced, dose escalation of the TKI being used is often possible.

Some guidelines, including BC Cancer Agency guidelines, include drug dose reduction to alleviate severe drug reactions. While side effects of the TKIs are often unpleasant, effort must be made to maintain patients on their cancer therapies. If a TKI is administered in only one dose, for example, in the case of gefitinib where only one dose exists, switching the patient to another TKI that has more flexible dosing is strongly recommended.

Dry skin in the trunk and extremities is very common in patients being treated with EGFR–TKIs. Fragrance free creams and ointments are recommended over lotions, which may contain alcohol. For scaling and hyperkeratosis, ammonium lactate and urea-containing preparations are also useful, but they should be used with care because of greater skin sensitivity in these patients.

A scalp rash may be successfully treated with the basic principles above, however, a gel can be formulated as cream and lotion treatment can be unappealing in the hair or hairline area of the neck. Patients may often develop lesions and plaques on the scalp, which can be treated with topical clindamycin 2% plus triamcinolone acetonide 0.1% in equal parts of propylene glycol and water until resolution.

## Musositis/Stomatitis

In addition to diarrhea, patients taking EGFR–TKIs often experience other gastrointestinal side effects. Although used interchangeably, mucositis refers to inflammation of the gastrointestinal tract while stomatitis refers to inflammation of the mouth (15, 16). Symptoms can also include tingling in the mouth or on the tongue, ulcers or cracks on the side of the mouth (B. Melosky, unpublished observation). This side effect is rarely seen with the first generation TKIs, but is observed with the second generation TKIs. In the original IPASS trial, 0.2% of patients treated with gefitinib reported grade 3 mucositis/stomatitis (11), while 8.7% of patients treated with afatinib in LUX-Lung 3 experienced grade 3 mucositis/stomatitis (Table 3) (13). As this is a new finding, the true incidence of mucositis/stomatitis has not often been evaluated in a trial setting.

### Management recommendations for mucositis/stomatitis

There have not been any randomized control trials to determine the best ways to manage EGFR–TKI induced mucositis/stomatitis. A careful examination of patients prior to treatment to determine baseline oral health status and inflammation is recommended, as well as observation during treatment (17). Patients are encouraged to practice good oral hygiene including frequent brushing with a soft brush, flossing, and rinsing with saline. Avoid commercial mouthwashes as they often contain alcohol, which can exacerbate the situation. As symptoms become more severe, oral care should become more frequent (16).

The following strategies for treating stomatitis/mucositis are recommended (Dr. Kim Papp, personal communication). The recommended treatment for grade 1 stomatitis/mucositis is kenalog in Orabase^®^, applied two or three times a day. The recommended treatment for grade 2 stomatitis/mucositis is kenalog in Orabase^®^, with the addition of 250–350 mg of erythromycin a day. The recommended treatment for grade 3 stomatitis/mucositis is clobetasol ointment instead of kenalog in Orabase^®^, with an increase in erythromycin dose to 500 mg daily. EGFR–TKI is maintained for grades 1 and 2, and temporarily discontinued for grade 3, until the stomatitis/mucositis improves to grade 2, at which point it is resumed at 50% of the original dose and then increased if symptoms do not get worse.

## Paronychia

In addition to the skin rash, other cutaneous manifestations may be observed in patients treated with EGFR–TKIs. Paronychia inflammation or infections associated with the lateral nail folds of the toes and fingers can become a concern after a longer period of treatment. Although EGFR–TKI related nail changes are usually mild, they can also be severe and symptomatic, especially with the newer generation of TKIs (8, 18). Of note, paronychia is almost never seen with first generation EGFR–TKIs, erlotinib, and gefitinib (Table 3).

### Management recommendations for paronychia

A challenge in managing paronychia is that there have not been any randomized control trials testing out treatment/management options. Patients are advised to use emollient lotions, to wear gloves during chores or cleaning, and to avoid impacts on fingers and toes.

The following strategies for treating the inflammation and infection are recommended (Table 5, Dr. Hirsh). The recommended treatment for grade 1 paronychia is topical antibiotics and antiseptics including clindamycin 1%, erythromycin 1%, tetracycline 1%, or chloramphenicol 1%, iodine ointment. Vinegar soaks, whereby fingers or toes are soaked in a 1:1 solution of white vinegar and water for 15 min a day are recommended. In addition, oral doxycycline may be effective along with a high potency corticosteroid such as clobetasol propionate applied to nail beds twice a day. For more severe cases (grade 2), silver nitrate may be applied weekly. Patients with splinter hemorrhages can be treated with liquid bandage. In severe cases (grade 3), the EGFT–TKI should be discontinued until symptoms improve. Additionally, intralesional corticosteroid injections or removal of the nail plate may be beneficial.

**Table 5:** **Management guidelines for paronychia (Hirsh, personal communication)**.

Grade	Toxicity	EGFR inhibitor
1	• Nail fold edema or erythema • Disruption of the cuticle	• Topical antibiotics/antiseptics^a^ • Vinegar soaks^b^ • Topical ultrapotent steroids (clobetasol priopionate) applied twice daily
2	• Nail fold edema or erythema with pain • Associated with discharge or nail plate separation • Limiting instrumental activities of daily living	• Same as in grade 1 • Silver nitrate application weekly
3	• Limiting self-care activities of daily living	**Interrupt afatinib; refer to a dermatologist and resume afatinib at a reduced dose (10 mg) if patient recovers to grade ≤1**
		• Same as in grade 2 • Consider nail avulsion and systematic antibiotics^c^

*^a^Examples of topical antibiotics/antiseptics: clindamycin 1%, erythromycin 1%, tetracycline 1%, or chloramphenicol 1%, iodine ointment*.

*^b^Vinegar soaks consist of soaking fingers or toes in a solution of white vinegar or water 1:1 for 15 min every day*.

*^c^Systemic antibiotics include tetracyclines and antimicrobials (erythromycin should be avoided)*.

## Conclusion

Tyrosine kinase inhibitors against the epidermal growth factor have become standard of care in cancers such as NSCLC. Therapy with EGFR–TKIs has improved clinical outcomes, but they are accompanied by a number of adverse events that can be effectively managed, especially diarrhea, rash, mucositis, and paronychia. Strategies for early and ongoing management of rash and diarrhea are essential to patient compliance and treatment outcome.

## Conflict of Interest Statement

The authors declare that the research was conducted in the absence of any commercial or financial relationships that could be construed as a potential conflict of interest.

## Abbreviations

BCCA, BC cancer agency; EGFR, epidermal growth factor receptor; NCI, National Cancer Institute; NSCLC, non-small cell lung cancer; QOL, quality of life; RCT, randomized clinical trial; SAE, severe adverse event; TKI, tyrosine kinase inhibitor.
